# The structural–functional-connectivity coupling of the aging brain

**DOI:** 10.1007/s11357-024-01106-2

**Published:** 2024-03-06

**Authors:** Hui Zhang, Peng Cao, Henry K. F. Mak, Edward S. Hui

**Affiliations:** 1 Department of Rehabilitation Sciences, The Hong Kong Polytechnic University, Hong Kong, China; 2 Research Institute for Smart Ageing, The Hong Kong Polytechnic University, Hong Kong, China; 3 Research Institute for Intelligent Wearable Systems, The Hong Kong Polytechnic University, Hong Kong, China; 4 Department of Diagnostic Radiology, The University of Hong Kong, Hong Kong, China; 5 Alzheimer’s Disease Research Network, The University of Hong Kong, Hong Kong, China; 6 State Key Laboratory of Brain and Cognitive Sciences, The University of Hong Kong, Hong Kong, China; 7 Department of Imaging and Interventional Radiology, The Chinese University of Hong Kong, Hong Kong, China; 8 Department of Psychiatry, The Chinese University of Hong Kong, Hong Kong, China; 9 CU Lab for AI in Radiology (CLAIR), The Chinese University of Hong Kong, Hong Kong, China

**Keywords:** Structural–functional-connectivity coupling, Small vessel disease, Aging, Cognitive reserve, Harvard Aging Brain Study

## Abstract

Aging primarily affects memory and executive functions, a relationship that may be underpinned by the fact that almost all adults over 60 years old develop small vessel disease (SVD). The fact that a wide range of neuropathologies could only explain up to 43% of the variation in age-related cognitive impairment suggests that other factors, such as cognitive reserve, may play a role in the brain’s resilience against aging-related cognitive decline. This study aims to examine the relationship between structural–functional-connectivity coupling (SFC), and aging, cognitive abilities and reserve, and SVD-related neuropathologies using a cohort of *n* = 176 healthy elders from the Harvard Aging Brain Study. The SFC is a recently proposed biomarker that reflects the extent to which anatomical brain connections can predict coordinated neural activity. After controlling for the effect of age, sex, and years of education, global SFC, as well as the intra-network SFC of the dorsolateral somatomotor and dorsal attention networks, and the inter-network SFC between dorsolateral somatomotor and frontoparietal networks decreased with age. The global SFC decreased with total cognitive score. There were significant interaction effects between years of education versus white matter hyperintensities and between years of education versus cerebral microbleeds on inter-network SFC. Enlarged perivascular space in basal ganglia was associated with higher inter-network SFC. Our results suggest that cognitive ability is associated with brain coupling at the global level and cognitive reserve with brain coupling at the inter-functional-brain-cluster level with interaction effect from white matter hyperintensities and cerebral microbleed in a cohort of healthy elderlies.

## Introduction

The human brain is made up of a vast and complicated network of anatomical connections (i.e., structural brain network) that permits efficient communication between different parts of the brain (i.e., functional brain connections or network) for segregation and integration of neural information [1]. Because of the strong correspondence between structural and functional brain networks [2, 3], recent efforts have focused on devising new biomarkers that can jointly characterize these two types of networks in the hope of improving our understanding on the relationship between brain versus behavior and cognition [4, 5]. One of the potential biomarkers is the structural–functional-connectivity coupling (SFC), which is estimated from the correlation between structural and functional connections [2, 6, 7]. It reflects the extent to which anatomical brain connections can predict coordinated neural activity, or the extent to which anatomical connections support brain communications [4].

The SFC of healthy youth [4], young adults [5], and patients with neuropsychiatric diseases and disorders [6, 8–14] has been investigated. In healthy participants, the regional pattern of SFC of both adolescent [4] and young adult brain [5] follows the cortical hierarchies, whereby unimodal sensory areas exhibit high coupling, and transmodal association areas exhibit low coupling. Significant association between regional SFC versus age and cognition was also demonstrated [4, 5].

Compared to healthy controls, decrease in SFC at the level of the whole brain were observed in patients with epilepsy [2, 6, 7], bipolar disorder [8], multiple sclerosis [11], Alzheimer’s disease [10], and Parkinson’s disease [9], whereas increase in SFC at the level of the whole brain was observed in participants with cognitive impairment with no dementia [13]. At the level of functional brain clusters (i.e., a group of functionally similar brain regions) and rich-club connections, increase in SFC was observed in the default-mode network and rich-club connections of patients with Alzheimer’s disease [14], and the non-rich-club connections of patients with mild cognitive impairment and Alzheimer’s disease [12], whereas decrease in SFC was observed in the salience, visual, and somatomotor networks of patients with multiple sclerosis [11]. At the level of individual brain regions, decrease in SFC was observed in the hippocampus, insula, frontal gyrus, and middle temporal gyrus of patients with Alzheimer’s disease [14], and the bilateral superior and middle occipital gyri, and right cuneus, precuneus, and calcarine gyrus of patients with Parkinson’s disease [9]. On the other hand, several studies demonstrated the association between SFC and cognition. Higher whole brain SFC was associated with better cognitive functions in patients with mild cognitive impairment and Alzheimer’s disease [12] and worse cognitive functions in participants with cognitive impairment with no dementia [13]. Higher SFC of right calcarine was correlated with better cognitive functions for patients with Parkinson’s disease [9]. Taken together, the pattern of change in SFC depends not only on the disease cohort but also on the scale at which coupling was measured.

Aging primarily affects memory and executive functions [15]. This connection may be underpinned by the fact that almost all adults over 60 years old develop small vessel disease (SVD), a common cause of stroke and vascular dementia that was previously considered innocuous [16, 17]. Common neuropathologies of SVD are white matter hyperintensities (WMH), lacunes, enlarged perivascular space (ePVS), and cerebral microbleeds (CMBs) [17]. Previously, the neuropathologies of Alzheimer’s disease, infarcts, and Lewy bodies were also thought to be the primary cause of age-related cognitive decline [18]. However, it is subsequently demonstrated that a wider array of neuropathologies, including markers of Alzheimer’s disease, limbic predominant age-related TDP-43 encephalopathy, hippocampal sclerosis, Lewy bodies, macroscopic infarcts, microinfarcts, cerebral amyloid angiopathy, atherosclerosis, and arteriolosclerosis, could only account for up to 43% of the variation in age-related cognitive impairment [19]. These findings suggest that alternative factors, such as the concept of cognitive reserve, brain reserve, and brain maintenance [20], may underpin the resilience of the brain to aging-related cognitive decline. Of note is that years of education, a proxy of cognitive reserve, is a potential modifiable risk factor for SVD-related cognitive decline [16, 21].

Considering the intricate link between aging, cognitive abilities, SVD-related neuropathologies and cognitive reserve, the primary aim of this study was to examine the degree to which SFC varied with these characteristics in a cohort of healthy elders from the Harvard Aging Brain Study (HABS) [22]. The SFC of the entire brain and that of the intra- and inter-functional-brain-clusters was investigated.

## Methods

### Participants

Demographic (e.g., age, sex, and education), clinical (e.g., Preclinical Alzheimer Cognitive Composite-96, PACC96) [23], and 3 T MRI (T1, T2, FLAIR and SWI images, diffusion, and resting-state fMRI) data were obtained from the HABS cohort of *n* = 284 healthy elders (age 62 to 92 years). The informed consent for all participants was obtained by HABS, and our data usage was approved by HABS [22].

Cohort-specific inclusion criteria for recruitment included being 62 years or older, obtaining a score of 0 on the Clinical Dementia Rating Scale, scoring over 25 on the Mini-Mental State Examination, achieving scores above the thresholds adjusted for age and education on the 30-Minute Delayed Recall of the Logical Memory Story A, and getting a score lower than 11 on the Geriatric Depression Scale. The criteria for exclusion encompassed a history of alcoholism, drug abuse, head trauma, or a current serious medical/psychiatric illness. [22]. The participants diagnosed with mild cognitive impairment and dementia were excluded from all analyses. For all statistical analysis of SFC, we used the subset of *n* = 176 participants with both the first year and 4th year follow-up data. For the GLM analysis, all *n* = 176 participants with the data from two visits, *n* = 44 participants with the first-year data only, and *n* = 7 participants with the 4th year follow-up data only were used.

### Small vessel disease (SVD) scoring

The scoring system for SVD used in this study has been well-established by Staals et al. [24]. The SVD scoring was conducted by consensus of a panel comprising one neuroradiologist (HKFM) and one neuroscientist (HZ). Lacunes manifest as round or ovoid, subcortical fluid-filled cavity and appear as a central CSF-like hypointensity with a surrounding rim of hyperintensity on FLAIR. Occasionally, other sequences such as T1-weighted and T2-weighted MRI are required for discerning lacunes when there is no hyperintense rim on FLAIR [25]. White matter hyperintensities are common in older adults and characterized by T2-weighted MR images. Deep and periventricular WMH were scored using the Fazekas scale between 0 and 3 [26]. Enlarged perivascular space (ePVS) is defined as small, sharply delineated structures of CSF intensity (< 3 mm following the tract of perforating vessels) that can be easily seen on T2-weighted images [27]. Cerebral microbleeds are defined as small (< 5 mm), homogeneous, round foci in the brainstem, white matter, cortico-subcortical junction, basal ganglia, and cerebellum on SWI images [24].

The SVD score is the total of four closely correlated features: lacunes, WMH, CMBs, and ePVS in basal ganglia. It is on an ordinal scale from 0 to 4. One point is given for each of the following: the presence of lacunes and CMBs was defined as the presence of one or more lacunes (1 point if present) or CMB (1 point if present); the presence of moderate to severe (grade 2–4) ePVS in the basal ganglia (1 point if present); and the presence of either confluent deep WMH (Fazekas score 2 or 3) or irregular periventricular WMH extending into the deep white matter (Fazekas score 3) (1 point if present) [24].

### Cognitive outcome

Participants in the HABS were evaluated annually with a battery of cognitive assessments. For this study, we evaluated cognition using the Preclinical Alzheimer Cognitive Composite (PACC-96), a mean of z score performances on four tests sensitive to cognitive decline in at-risk individuals: mini-mental state examination, logical memory, digit symbol coding, and the free and cued selective reminding test (sum of free recall and total score added together) [28].

### Construction of the structural connectomes

HABS participants underwent MRI examination using a 3 T MR scanner (Siemens Tim Trio) with a 12-channel head coil. Structural images were acquired with 3D T1-weighted sequence using magnetization prepared rapid gradient echo imaging (MPRAGE, 256 sagittal slices, repetition time (TR) = 2300 ms, echo time (TE) = 2.95 ms, inversion time (TI) = 900 ms, flip angle (FA) = 9°, FOV = 270 × 253 mm, matrix = 256 × 240, voxel size = 1.05 × 1.05 × 1.2 mm). The diffusion tensor images (DTI) were acquired using spin-echo echo-planar imaging with TR/TE of 3900/81 ms, 2 × 2 × 2 mm^3^ voxel size, *b*-values of 1000 s/mm^2^, and 30 diffusion encoding directions.

We used the Pipeline for Analyzing braiN Diffusion imAges (PANDA, http://www.nitrc.org/projects/panda) to process the DTI data [29]. PANDA is a MATLAB toolbox for automated processing of brain diffusion images based on the Diffusion Toolkit (http://www.trackvis.org/dtk/) and the fMRI Software Library (FSL, http://fsl.fmrib. ox.ac.uk/fsl). Firstly, the DTI data were corrected for motion and eddy current geometric distortions, and non-brain tissues were removed. Participants with head movements of more than 3 mm in any direction of *x*, *y*, *z*, or over 3° were excluded. The fractional anisotropy (FA) of each voxel was subsequently estimated. The FA images were then coregistered with T1 image in the native space. Probabilistic tractography was subsequently performed using ProbtrackX of FSL (Fibers = 3, weight = 1, burnin = 1000). The Gordon brain parcellation [30] in the standard Montreal Neurological Institute (MNI) space were warped to the individual’s native space by the inverse transformations of image normalization and coregistration. Each region of the brain was defined as a brain network node. Finally, a 333 × 333 structural connectivity matrix (SC) was estimated by counting the number of streamlines between all pairs of brain parcels.

### Construction of the functional connectomes

Resting-state fMRI data were acquired using gradient-echo echo-planar sequence with TR/TE = 3000/30 ms, flip angle = 90°, voxel size = 3 × 3 × 3 mm^3^. The analysis of fMRI data was performed using the Data Processing Assistant for Resting-State fMRI (DPARSF) and Statistical Parametric Mapping (SPM12). The first 10 volumes were discarded, and the differences in image acquisition time of the remaining fMRI images were corrected. Next, the time series of images for each participant were realigned using a six-parameter linear transformation with a two-pass procedure (registered to the first image and then registered to the mean of the images after the first realignment). Participants with head motion more than 3 mm in *x*, *y*, and *z* or 3° were excluded. In addition, the Diffeomorphic Anatomical Registration Through Exponentiated Lie algebra (DARTEL) tool [31] normalized the structural images and tissue maps, which were obtained from structural images, to Montreal Neurological Institute (MNI) space and created transformation parameters. Mean WM and CSF (from tissue maps) time series were regressed out from the time course in each voxel. All the fMRI images were spatially normalized to the MNI space and resampled to $3\times 3\times 3$ mm^3^ using the transformation parameters that were estimated through DARTEL segmentation. After the normalization, the data were band-pass filtered (0.01–0.08 Hz) to reduce high-frequency respiratory and cardiac noise and low-frequency drift. For each participant, the regional time series was obtained by averaging the time series over all voxels of each of the parcel in the Gordon parcellation. After retrieval of the mean individual regional time series, we calculated the correlation coefficients between all pairs of brain parcels to obtain a 333 $\times$ 333 functional connectivity (FC) matrix for each participant. We applied the Fisher *z*-Transformation to the correlation coefficients so that all FC became normally distributed.

### Functional brain clusters

**Fig. 1:**
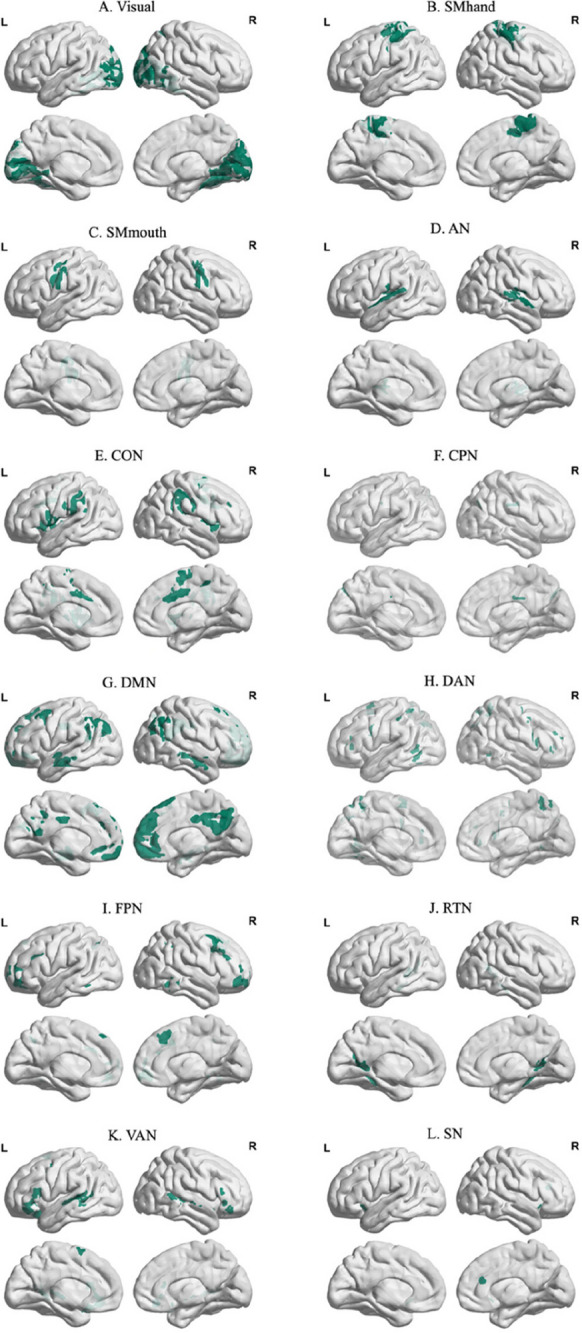
Spatial distribution of network modules. The 333 brain regions from the Gordon atlas were aligned to twelve different functional brain clusters [30], namely, **A** visual (VIS), **B** dorsal somatomotor (SMhand), **C** ventral somatomotor (SMmouth), **D** auditory network (AN), **E** cingulo-opercular network (CON), **F** cingulo-parietal network (CPN), **G** DMN, **H** dorsal attentional network (DAN), **I** frontoparietal network (FPN), **J** retrosplenial temporal network (RTN), **K** ventral attentional network (VAN), and **L** salience network (SN)

Individual brain parcels can be assigned to specific functional brain clusters that have been implicated in different cognitive functions. In this study, we used the Gordon parcellation with 12 functional brain clusters (Fig. 1), namely, the visual (VIS), dorsal somatomotor (SMhand), ventral somatomotor (SMmouth), auditory (AN), cingulo-opercular (CON), cingulo-parietal (CPN), default mode (DMN), dorsal attentional (DAN), frontoparietal (FPN), retrosplenial temporal (RTN), ventral attentional (VAN), and salience (SN) networks [30]. These functional brain clusters or networks will be used for subsequent intra and inter-network SFC and modularity (see below).

### Intra and inter-network modularity

The modularity of structural and functional brain clusters were computed using the same method using the GRETNA toolbox (https://www.nitrc.org/projects/gretna/) [32]. Of note is that we used the Gordon’s 12 functional brain clusters as *a prior* modules for estimating the intra and inter-network modularity, instead of the data-driven method used in the original study by Newman.

### Estimation of SFC

**Fig. 2:**
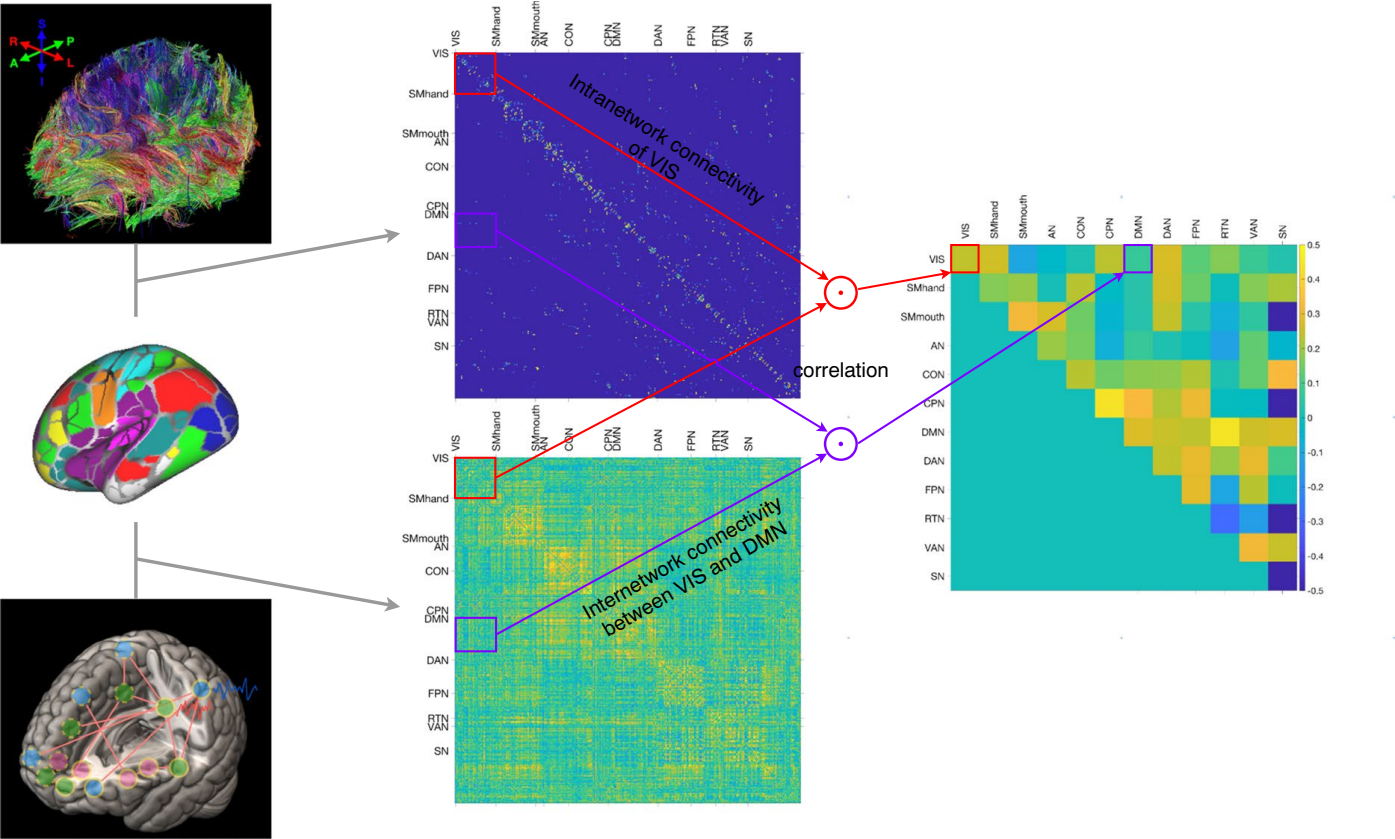
Illustration on the estimation of SFC at intra and inter-functional-brain-cluster levels. Intra-network SFC was computed from correlation between the structural and functional connections of brain regions within a single functional brain cluster, whereas inter-network SFC from correlation between the structural and functional connections of brain regions from a pair of different functional brain clusters

The global SFC was estimated by calculating the Spearman-rank correlation between the entire SC matrix with the FC matrix, similar to the algorithm previously described by Gu et al. [5]. The intra and inter-functional-brain-network SFC were also computed using the 12 functional brain clusters from the Gordon template [30]. For intra-network SFC, the SC and FC of brain regions within individual functional brain clusters were correlated. For inter-network SFC, the SC and FC between brain regions from different functional brain clusters were correlated (see Fig. 2 for overview).

### Statistical analysis

To investigate the relationships between $\overline{Q }$, SFC versus age, sex, years of education (edu), PACC96, and individual SVD lesion type (WHM, lacunes, ePVS, and CMBs), the following linear mixed model was performed using R:

$$\mathrm{Imer}\;\left(\mathrm{SFC}\;\sim\;1+\;\mathrm{sex}\;+\;\mathrm{age}^\ast\mathrm{PACC}96+\mathrm{edu}^\ast\mathrm{PACC}96\;+\;\mathrm{age}^\ast\mathrm{WMH}\;+\;\mathrm{edu}^\ast\mathrm{WMH}\;+\;\mathrm{age}^\ast\mathrm{lacunes}+\;\mathrm{edu}^\ast\mathrm{lacunes}\;+\;\mathrm{ag}e^\ast\mathrm{ePVS}\;+\;\mathrm{edu}^\ast\mathrm{ePVS}+\mathrm{age}^\ast\mathrm{CMBs}+\mathrm{edu}^\ast\mathrm{CMBs}+\;\left(1/\mathrm{participant}\right)\right)$$

All the SVD lesion types and sex are within-participant categorical fixed factors, and edu and age are within-participant continuous fixed factors. Participant is a random factor.

To investigate the change in the brain network (intra and inter-network modularity) and SFC between the first and second visits after accounting for the effect of age, sex, and years of education, within-subject anova with covariate was performed using SPSS (SPSS, Inc., Chicago, IL, USA). All *p* values were FDR corrected for multiple comparisons. A significance level was set at *p* < 0.05 for all statistical tests.

## Results

Table 1 summarizes the demographics and clinical variables of all healthy elders. The global SFC significantly decreased, and PACC96 significantly increased in the second visit after accounting for the effect of age, sex, and years of education. The number of healthy participants with WMH and ePVS increased, respectively. The total SVD score also increased.

**Table 1:** Demographics and clinical variables of a cohort of healthy elders from the HABS

Characteristics		Participants scanned in both visits		*p* value	Participants scanned in one visit only
Number		176		-	51
Sex (F/M)		111/65		-	31/20
Edu		16.1 ± 3.0		-	15.5 ± 3.1
		Visit 1	Visit 2		One visit
Age		73.1 ± 6.1	76.2 ± 6.1	** < 0.001**	74.1 ± 5.8
Global SFC		0.193 ± 0.043	0.187 ± 0.041	**0.013**	0.185 ± 0.045
PACC96		0.11 ± 0.59	0.25 ± 0.71	** < 0.001**	− 0.28 ± 0.86
		Number			
Lacunes	N	149	150	-	40
	Y	27	26	-	11
WMH	N	76	58	**-**	20
	Y	100	118	**-**	31
ePVS	N	73	57	**-**	23
	Y	103	119	**-**	28
CMBs	N	135	132	-	41
	Y	41	44	-	10
SVD score	0	36	23	**-**	10
	1	49	45	**-**	15
	2	57	65	**-**	15
	3	29	39	**-**	9
	4	5	4	**-**	2

*Edu*, years of education; *WMH*, white matter hyperintensities; *ePVS*, enlarged perivascular space; *CMBs*, cerebral microbleeds; *SVD*, small vessel disease; *PACC96*, Preclinical Alzheimer Cognitive Composite-96; *N*, no; *Y*, yes

Values in bold indicate significant difference between the 2 visits

**Fig. 3:**
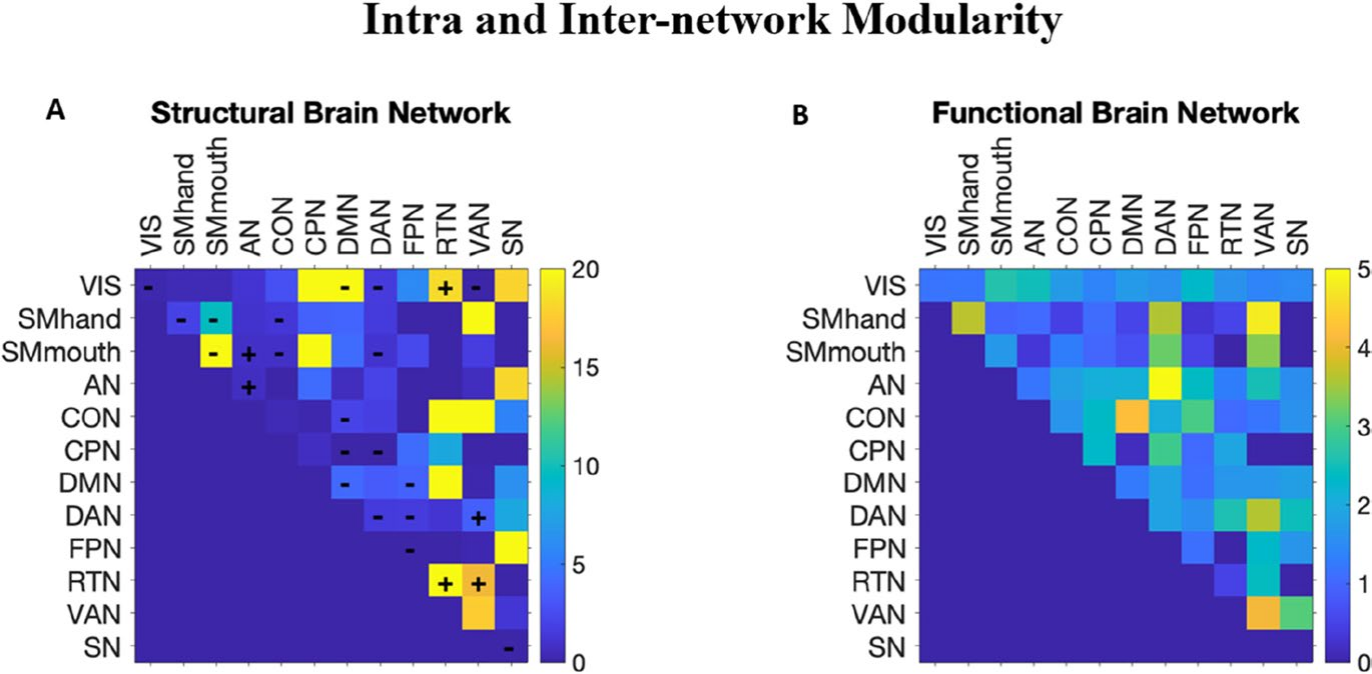
The *F*-statistics for the comparison in the intra and inter-network modularity of the structural (**A**) and functional (**B**) brain network of healthy elderly between the first and second visit. Significant increase in modularity was indicated using + and decrease using − . VIS, visual; SMhand, dorsal somatomotor; SMmouth, ventral somatomotor; AN, auditory network; CON, cingulo-opercular network; CPN, cingulo-parietal network; DMN, default mode network; DAN, dorsal attentional network; FPN, frontoparietal network; RTN, retrosplenial temporal cortex; VAN, ventral attentional network; SN, salience network

Figure 3A illustrates the difference in the modularity of structural brain network between the two visits (second visit–first visit). After accounting for the effect of age, sex, and years of education, significant decrease in intra-network modularity of the VIS, SMhand, SMmouth, DMN, DAN, FPN, and SN and increase in that of the AN and RTN were observed. Significant decrease in the inter-network modularity between VIS × DMN, VIS × DAN, VIS × VAN, SMhand × SMmouth, SMhand × CON, SMmouth × CON, SMmouth × DAN, CON × DMN, CPN × DMN, CPN × DAN, DMN × FPN, and DAN × FPN was observed. Significant increase in the inter-network modularity between VIS × RTN, SMmouth × AN, DAN × VAN and RTN × VAN was observed. No significant difference was found in the modularity of functional brain network (Fig. 3B).

**Fig. 4:**
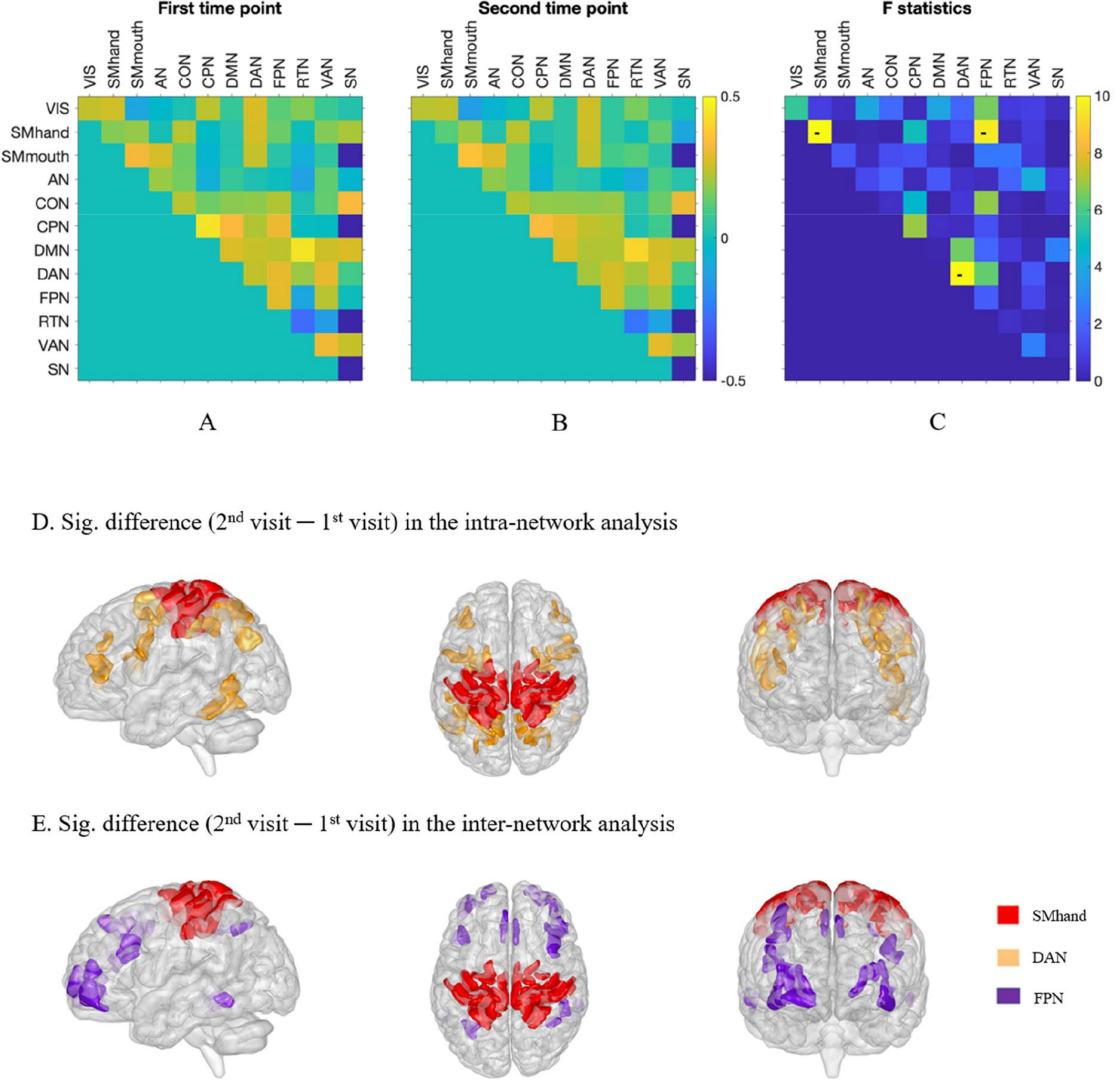
Group-averaged intra and inter-network coupling **A** at the first visit (*N* = 176) and **B** at the second visit (3 years apart) (*N* = 176) **C** shows the *F*-statistics for the comparison in the intra and inter-network coupling. Significant decrease in SFC was indicated using − . **D**, **E** The brain regions of the functional brain clusters with significant difference in SFC from baseline. All *p* values were FDR-adjusted

Figure 4 shows the group-averaged intra- and inter-network SFC measured at the two visits (3 years apart), and the difference in coupling between the two visits (second visit–first visit). After accounting for the effect of age, sex, and years of education, significant decrease in the intra-network SFC of SMhand and DAN (Fig. 4D) and the inter-network SFC between SMhand × FPN were observed (Fig. 4E).

Table 2 presents the significant relationships between SFC versus participant characteristics. A negative correlation between cognition (PACC96) and global SFC was observed (${\chi }^{2}\left(1\right)=5.02$, *p*-value = 0.0251). The inter-network SFC of the RTN × CPN of participants with ePVS was higher than those without (${\chi }^{2}\left(1\right)=14.06$, *p*-value = 0.0119). The WMH has an interaction effect on the association between years of education and the inter-network coupling between DMN and SN (${\chi }^{2}\left(1\right)=13.37$, *p*-value = 0.0171). Higher years of education was trending towards lower inter-network SFC between DMN and SN for participants without WMH, but higher SFC for those with WMH. The CMBs has an interaction effect on the association between years of education and the inter-network coupling between CON and SN (${\chi }^{2}\left(1\right)=18.53$, *p*-value = 0.0011). Higher years of education were trending towards higher inter-network SFC between CON and SN amongst participants with CMBs.

**Table 2:** Effects of demographics and clinical variables including age, sex, years of education, PACC96, and individual SVD scores (lacunes, WMH, CMBs, and ePVS) on the coupling between structural and functional brain clusters

	Linear mixed model^#^					Likelihood ratio	
	*β*	S.E	df	*t*-value	*p* value*	${\chi }^{2}(1)$	*p* value*
Global SFC							
PACC96	− 0.005	0.002	339	− 2.204	0.028	5.02	0.025
Inter-network SFC							
RTN × CPN							
ePVS	0.184	0.052	98	3.562	0.038	14.06	0.012
DMN × SN							
Edu × WMH	0.086	0.024	316	3.601	0.025	13.37	0.017
CON × SN							
Edu × CMBs	0.112	0.027	250	4.232	0.002	18.53	0.001

*S.E.*, standard error; *df*, degree of freedom; *CON*, cingulo-opercular; *CPN*, cingulo-parietal network; *DMN*, default mode network; *RTN*, retrosplenial temporal network; *SN*, salience network

^#^Data from all participants (regardless of whether he/she came for the second visit) have been used in this statistical analysis

^*^p values were FDR-corrected (except global SFC)

## Discussion

### The relationship between SFC versus age

In a cohort of healthy elders, after controlling for the effect of age, sex, and years of education, we have shown that the global SFC (see Table 1), as well as the intra-network SFC of the dorsolateral somatomotor and dorsal attention networks, and the inter-network SFC between dorsolateral somatomotor and frontoparietal networks decreased with age (see Fig. 4C). Our results largely corroborated with those of a recent study of SFC across lifespan, wherein they have shown that this negative correlation pattern existed not only globally but also within the somatomotor systems [33]. Of note is that the major discrepancy between our findings and those of Zamani-Esfahlani et al. [33] pertains to the fact that we observed age-related decreases in the SFC of the DAN and FPN and that we did not find age-related decrease in the SFC of VIS. This discrepancy may be caused by the difference in age ranges between the two datasets.

In another study of healthy aging, aging-related change in the functional connectivity of the somatosensory and dorsal attention networks were also observed [34]. In a different study of cognitive reserve in healthy aging, elders with high cognitive reserve were shown to have different functional connectivity of dorsal attention and frontoparietal networks compared to elders with low cognitive reserve, and these differences were correlated with better cognition [35]. Taken together, these results suggest that changes in the constraint of brain communication by structural connections in the dorsolateral somatomotor, dorsal attention, and frontoparietal networks may likely help to compensate for the effect of aging on brain functions.

### The relationship between SFC versus cognition

We have shown that the SFC at the level of whole brain, but not individual functional brain clusters, decreased with higher cognitive score in healthy elders (see Table 2). Several prior studies have also demonstrated association between global brain coupling versus brain function and behavior. Wang et al. have shown that global SFC also decreased with cognition in a cohort of elders with cognitive impairment and similar ages to those of our study. On the contrary, Medaglia et al. have observed that the global alignment between structural and functional connectivities increased with cognitive flexibility in a cohort of young adults [36]. Taken the results from studies of normal and cognitively impaired elderlies together, lower global brain coupling was associated with higher cognitive functions. These results likely suggest that the cognition in healthy elderlies may be supported by cognitive processes that are less constraint by structural connections at the global level, while those with worse cognitive abilities have more tethered structure–function associations [37, 38].

At the level of local regions, the relationship between coupling and cognition is varied. Higher executive function was associated with higher SFC of the rostro-lateral frontal and medial occipital regions, and lower executive function with higher SFC of the primary motor cortex in youth [4]. Total cognition score was shown to be positively correlated with the SFC of anterior insula/putamen, and negatively correlated with the SFC of middle cingulate and supplementary motor areas of young adults [5]. Together with our findings on the lack of association between total cognitive score and the SFC of functional brain clusters, these results likely indicated that the brain structure of the individual brain regions of youth and young adult can flexibly constrain functional connections for supporting different cognitive functions, whereas the cognitive functions of elderlies are supported by minimal variations in the structure–function association of individual functional brain clusters.

### Modulation on the relationship between years of education and brain coupling by white matter hyperintensities and cerebral microbleeds

Our results have demonstrated that there were significant interaction effects by WMH and CMBs on the relationship between years of education versus inter-network SFC, but no significant linear effect between years of education and inter-network SFC (see Table 2). More specifically, higher education was trending towards lower inter-network SFC between DMN and SN for participants without WMH, but higher SFC for those with WMH. On the other hand, there was a positive trend between years of education and the inter-network SFC between CON and SN amongst participants with CMBs. In other words, in the presence of either of the two SVD neuropathologies, healthy elders with higher education may tend to have higher inter-network SFC. Benson et al. demonstrated that the functional connectivity of SN significantly moderated the impact of WMH on executive functions in a cohort of healthy elders and patients with mild cognitive impairment [39]. In another study of healthy elders, high functional connectivity of SN was associated with higher education and better cognitive functions [40]. Considering that cognitive reserve, of which years of education is a proxy [21], has a protective effect against aging-related cognitive decline [21] and SVD-related clinical manifestations [41–43], these results, together with ours, likely suggest that stronger alignment of functional activation patterns to the underlying structural brain connections may be necessary for healthy elders with higher cognitive reserve to protect against the insidious effects of WMH and CMBs.

### Effect of enlarged perivascular space in basal ganglia on SFC

Our study demonstrated that healthy elders with ePVS in basal ganglia exhibited higher inter-network SFC between RTN and CPN than those without ePVS. Previous studies of healthy elderlies have demonstrated that age-related increase in the visibility of basal ganglia PVS was found primarily in elders [44]; that the burden of ePVS in basal ganglia positively correlated with the decline in language, information processing, executive function, and episodic memory over a 4.7-year follow-up of healthy elderlies [45] and that the retrosplenial cortex, part of the RTN, has been implicated in the episodic memory [46]. Taken together, our results suggest that higher SFC between RTN and CPN may be necessary to protect against the insidious effect of ePVS in basal ganglia on cognitive functions in healthy elders.

### The DTI and fMRI modularity

The study found that there was a significant difference in modularity of structural brain network, but not functional brain network, between two visits. This suggests that there may be alterations in the wiring or organization of white matter tracts, which could reflect changes in the anatomical basis of brain function. The lack of significant change in the modularity of functional brain network could potentially be attributed to compensatory mechanisms within the brain. Compensatory mechanisms refer to the brain’s ability to adapt and reorganize its functional networks in response to changes or damage in certain areas to maintain overall function. These findings highlight the importance of considering both structural (DTI) and functional (fMRI) aspects of brain imaging when studying brain alterations. While DTI may provide insights into the underlying anatomical changes, fMRI can provide information about functional connectivity and compensatory mechanisms that may not be evident in structural imaging alone. Integrating both modalities can offer a more comprehensive understanding of brain changes and their implications for brain function.

### Limitations

First, several approaches have been proposed to estimate the coupling between structural and functional brain connections, namely, statistical, biophysical, and communication models [47]. Esfahlani et al. have demonstrated that SFC estimated using correlation model could be improved using communication models [33, 47]. The major limitation of the correlative approach is that it is non-mechanistic and thus offers limited sight on the mechanism underlying SFC [48]. On the contrary, communication models could account for the flow of signal through the underlying structural connections [49]. Esfahlani et al. have demonstrated that the communication model that best explains the variance in functional connections depends on functional brain clusters (i.e., system-specific) [33]. We will therefore investigate the effect of demographics and clinical variables on the SFCs estimated from different models in future studies. Second, it is noteworthy that common SVD neuropathologies, such as WMH, CMBs, may not cause symptoms individually, but are associated with clinical manifestations, such as cognitive impairment and dementia, when there is increasing number and different combinations of individual lesion types [17]. Previous studies have also shown that cognitive reserve may protect against some of these SVD-related clinical manifestations, including cognitive decline [41], motor impairment [42], and age at stroke [43]. It was also postulated that cognitive reserve may underpin the individual difference in cognition albeit similar SVD burden [50]. Future studies of a larger cohort of elders should thus also investigate the relationship between SFC versus burden of individual SVD lesion type and cognitive reserve. Final limitation pertains to the fact that our findings are limited to the scale of whole brain and individual functional brain clusters, thereby precluding the ability to identify individual brain regions that are most susceptible to the effect of aging and SVD.

## Conclusion

Our results suggest that cognitive ability is associated with brain coupling at a global scale, while the relationship between cognitive reserve and the brain coupling at the inter-functional-brain-cluster level was modulated by white matter hyperintensities and cerebral microbleed in a cohort of healthy elderlies.
